# Epidemiological survey of early childhood caries in Cambodia

**DOI:** 10.1186/s12903-019-0800-y

**Published:** 2019-06-13

**Authors:** Bathsheba Turton, Tepirou Chher, Wael Sabbah, Callum Durward, Sithan Hak, Arnaud Lailou

**Affiliations:** 1 Melbourne Dental School, University of Melbourne, Melbourne, Australia; 2 Oral Health Bureau, Department of Preventive Medicine, Ministry of Health, Phnom Penh, Cambodia; 3 Kings College London, London, UK; 4 University of Puthisastra, Phnom Penh, Cambodia; 5 UNICEF-Cambodia, Phnom Penh, Cambodia

**Keywords:** Early childhood caries, Cambodia, Oral-health-related quality-of-life, Family impact scale

## Abstract

**Background:**

The Southeast Asian Forum for Early Childhood Caries identified the need for more epidemiological surveys involving preschool children. To date, the only data on Early Childhood Caries in Cambodia come from convenience samples and only using the basic dmft index without measurement of the early signs of disease.

**Methods:**

A cross-sectional survey on an epidemiological sample of Cambodian preschool children was conducted in conjunction with the fourth follow-up of the Cambodian Health and Nutrition Monitoring Study. Children were examined in a field setting using both the South East Asian Index for Early Childhood Caries as well as the ‘pulpally involved, ulcerated, fistula, abscess’ (pufa) index. Caregivers also participated in a short questionnaire covering dietary habits, oral health knowledge and behaviors, as well as the Family Impact Scale (FIS) for Oral-Health-Related Quality-of-Life.

**Results:**

The sample included 3985 participants between birth and 4-years of age, across three provinces. There was an even sex distribution (50.7% male). Overall 56.6% of participants had one or more carious lesions and 5.4% had one or more pulpally-involved teeth. There were some significant differences by age and location. Among those in the 3-year-old age group 84.9% had at least one decayed tooth, and 16.1% had one or more pulpally-involved teeth. There were differences in oral health knowledge and behaviors by province; those in Phnom Penh reported more favorable responses. Consumption of non-nutritious foods also differed between provinces with those in Phnom Penh consuming a higher mean number of sweet beverages per day. Those children with at least one pulpally involved tooth had a ten times greater chance of realizing an impact across the FIS.

**Conclusions:**

Cambodian preschool children have a severe burden of dental caries and a high proportion of families are impacted by this problem. There were differences in oral health knowledge and behaviors according to province and this translated into differences in caries experience. The data from this study support the need for urgent action to address the issue of ECC in Cambodia.

## Introduction

In 2016 the South East Asian (SEA) Forum for Early Childhood Caries identified the need for more epidemiological surveys of Early Childhood Caries in the region. In the past, most epidemiological surveys of dental caries in SEA have involved only children who were 6-years and older and did not record the presence of white spot lesions. This is an important omission given that white spot lesions represent both the latent burden of disease and also an opportunity for non-invasive interventions prior to cavitation [1]. Cambodia itself lacks any true epidemiological data on Early Childhood Caries. A number of initiatives grew out of the South East Asian Forum, including a published literature review [1] and a protocol for epidemiological surveys of ECC in SEA - the South East Asian Index for Early Childhood Caries (SEA-ECC) [2].

Within Cambodia there is a growing body of data pointing to the severity of caries experience among young children. The 2011 Cambodian National Oral Health Survey (CNOHS) reported a dmft of 9.0 for 6-year-old children, a caries prevalence of 97%, and a prevalence of pulpally-involved teeth of 86% [3]. This level of disease places Cambodia among the most severe in the world in terms of dental caries [1]. In addition, the CNOHS found that 99% of the carious lesions among children go untreated. Up until now the surveys on Early Childhood Caries in Cambodia have been based on convenience samples only, and have not included any data on quality of life; therefore it is difficult to convince the authorities to undertake country-wide action on the issue and to provide the level of evidence needed to inform policy [4–6].

The impacts of Early Childhood Caries are far reaching and can affect a child’s ability to sleep, eat and grow [7]. In addition, the symptoms of the disease also affect others in the child’s household who may have their sleep disrupted, or need to take time off work in order to seek treatment for the child [8]. Therefore, in addition to being a condition with high prevalence and severity with a range of negative impacts on the child, Early Childhood Caries in Cambodia is potentially creating a large and previously unmeasured impact on those in the child’s household. The aim of the present paper is to describe the epidemiology, and family impacts of Early Childhood Caries in Cambodia and lay out an agenda for further investigations.

## Methods

This cross-sectional study was conducted in combination with the on-going Cambodian Health and Nutrition Monitoring Study (CAHENMS). The focus of the CAHENMS is to observe the health, nutrition and diet of pregnant and lactating women along with their preschool children (< 5 years of age) across three provinces. These provinces were identified by UNICEF for Enhanced Health Monitoring over a 3-year period. The sample size calculation was based on the three key indicators of health for preschool children; stunting, diarrhoea, and acute respiratory infection. Lists of pregnant and lactating women, and their respective children were obtained from village health volunteers and midwives in the selected Operational Districts until the target sample size of 1500 was reached. At baseline in 2016, the study enrolled children aged less than 24 months and 3 target groups of women: women at reproductive age, lactating women and pregnant women. Data in the present investigation were collected as part of the fourth follow-up in the CAHENMS in August 2017. Child participants were recruited from the three provinces (Phnom Penh, Kratie, and Ratinakiri) as they were born into the target communes; the sample began with 1500 children per province.

Ethical approval was provided by the National Ethics Committee for Health Research, Ministry of Health, Cambodia. Consent was obtained in writing from the caregivers at baseline and again verbally prior to participation of the caregiver in the questionnaire and the child in the intra-oral examination.

### Clinical examination

Children were examined in a supine position with a mouth mirror and illumination from a hand-held torch. Appropriate infection control procedures were performed. Data were collected on caries status using the South East Asian Early Childhood Caries (SEA-ECC), and the PUFA index [9]. The SEA-ECC index has 10 codes; Code 0 represents a sound tooth surface, Code 1 represents early demineralisation such as a smooth white opacity or brown discolouration, Code 2 represents signs of early enamel breakdown (cavitation), Code 3 represents a where dentine is exposed, Code 4 is a filled and sound tooth, Code 5 represents a sealed and sound tooth, Code 6 represents a filled or sealed tooth with a visible carious cavitation, Code 7 represents a tooth that has been extracted due to caries, Code 8 represents and unerupted tooth, Code X represents a tooth that is to be excluded due to non-carious defects. A child was said to have ‘any caries’ if they had one or more teeth with a Code 1, Code 2, Code 3, Code 4, or Code 6, or Code 7. ‘d_1_mft’ represented the number of teeth with lesions that were code 1 or higher. A child was said to have ‘any cavitated lesions if they had one or more lesions of code 2 or higher. ‘d_2_mft’ represented the number of teeth that had lesions that were cavitated (code 2 or above). A child was said to have ‘any pufa’ if there were one or more pulpally-involved teeth as defined by the PUFA index [9].

The CAHENMS involved eight data collection teams in a field environment. These teams were supplemented by a two-person dental team. The dental team included one calibrated examiner and one trained assistant. There were eight senior dental students who acted as examiners and underwent calibration training until all achieved a kappa score above 0.9 for the SEA-ECC index indicating near perfect agreement. Children received a toothbrush, toothpaste, and an oral health information brochure upon completion of the intra-oral examination.

### The questionnaire

Questions were asked on diet, oral health knowledge and behaviours. For those in the Phnom Penh sample, the Family Impact Scale (FIS) was used as a measure of Oral-Health-Related Quality-of-Life (OHRQoL). The FIS has been previously validated for use in a Cambodian population [10]. The instrument includes 8 questions across three subscales: family activities, family emotions, and conflict. The response options for each item were “Never” (scoring 0), “Once or twice” (scoring 1), “Sometimes” (scoring 2), “Often” (scoring 3), or "Every day or almost every day (scoring 4). A participant was said to have an ‘impact’ in a particular domain if they responded with a score 3 or above in any item. The questions on diet related to the frequency of consumption of non-nutritious (junk) foods over the preceding 24 h. Interviewers underwent two training sessions prior to collecting data and a field supervisor was present or contactable throughout the data collection phase to answer any questions. The questionnaire was administered to the caregiver of each participating child. Questions on oral health knowledge and behaviours were asked as open-ended questions whereby participants were not prompted and had the opportunity to add as many responses as they choose. Responses were then categorised and those responses falling outside defined categories were considered as ‘other’. Questions on diet were asked separately for each food group and the responding caregiver was asked to recall the number of times that the food item was consumed across six indicator time periods over the previous 24 h.

### Data management and analysis

Data were collected and managed using the existing database systems employed by the CAHENMS study. The system being used was the “KoBo Toolbox” and data were entered directly onto tablets for uploading at a location where there was WiFi access. Data were drawn from the KoBo Toolbox data base in a Microsoft Excel spreadsheet format prior to being transferred to IBM-SPSS (Version 23) for analysis. Differences in means between groups were tested using the Kruskal-Wallis test for non-parametric data and *t*-test for parametric data; differences in proportions among groups were tested using the Chi-squared test. The level of statistical significance was set at a *P*-value < 0.05. Multivariate modelling was outside the scope of the present paper and will be presented as part of the reporting of the wider CANHEMS study.

## Results

There was an even spread in terms of age and sex, with no statistically significant differences between groups of participants in each province (Table 1).

**Table 1:** Sociodemographic characteristics of participants

	Male N (row %)	Female N (row %)	Total N (column %)
Province			
Phnom Penh	542 (48.1)	584 (51.9)	1126 (28.3)
Ratinakiri	780 (49.5)	797 (50.5)	1577 (39.6)
Kratie	663 (51.7)	619 (48.3)	1282 (32.2)
Age-group			
< 1-year	389 (49.6)	395 (50.4)	784 (19.7)
1-year	671 (48.9)	701 (51.1)	1372 (34.5)
2-years	719 (50.6)	703 (49.4)	1422 (35.7)
> 3-years	205 (50.7)	199 (49.3)	404 (10.1)
Overall	1985 (49.8)	2000 (50.2)	3985 (100.0)

There was a severe experience of caries across the sample. Although only around half the children overall had one or more carious lesions, five out of six in the 3-year-old age-group had “any caries” and one in six had one or more pulpally-involved teeth. There were significant differences in caries experience by sex, age, and province; females and those in Phnom Penh had an approximately 10% higher prevalence of caries than other groups. There appeared to be a gradient whereby each year of life was associated with an approximately 20% higher prevalence of of cavitated lesions (Table 2).

**Table 2:** Clinical status by sociodemographic characteristics

	Any caries N (Row %)	*p*-value	Any Cavitated Lesions N (Row %)	p-value	Any pufa N (Row %)	p-value	d_1_mft Mean (SD)	p-value	d_3_mft Mean (SD)	p-value	Pufa Mean (SD)	p-value^a^
Sex												
Male	1082 (54.5)	0.005	546 (27.5)	0.001	104 (5.2)	0.358	3.3 (4.4)	< 0.001	1.2 (2.6)	< 0.001	0.2 (0.8)	0.654
Female	1172 (58.6)		646 (32.3)		111 (5.6)		3.8 (4.7)		1.5 (2.9)		0.2 (1.0)	
Age group												
< 12-months	77 (9.8)	< 0.001	19 (2.4)	< 0.001	6 (0.8)	< 0.001	0.4 (1.7)	< 0.001	0.1 (1.0)	< 0.001	0.0 (0.7)	0.002
12–23 months	763 (55.6)		269 (19.6)		37 (2.7)		2.7 (3.4)		0.7 (1.7)		0.1 (0.5)	
24–35 months	1068 (75.1)		637 (44.8)		107 (7.5)		5.1 (4.9)		2.0 (3.1)		0.2 (1.0)	
36–47 Months	343 (84.9)		265 (65.6)		65 (16.1)		7.0 (5.6)		3.6 (4.1)		0.5 (1.7)	
Province												
Phnom Penh	626 (55.6)	0.005	387 (34.4)	< 0.001	66 (5.9)	0.307	3.7 (4.8)	0.008	1.7 (3.1)	0.018	0.1 (0.7)	0.181
Kratie	856 (54.3)		418 (26.5)		90 (5.7)		3.4 (4.6)		1.1 (2.6)		0.2 (1.2)	
Ratanakiri	772 (60.2)		387 (30.2)		59 (4.6)		3.6 (4.2)		1.3 (2.6)		0.1 (0.7)	
Total	2254 (56.6)		1192 (29.9)		215 (5.4)		3.5 (4.5)		1.3 (2.8)		0.2 (0.9)	

^a^*P*-values presented are χ^2^ tests for comparisons of proportions and the Kruskal-Wallis test for comparison of means among groups within the same column

Most of the participants believed that primary teeth were “important” although one-third of the participants did not believe that tooth decay is preventable (Table 3). Regarding methods for prevention, two-thirds knew that tooth brushing was a technique for preventing dental caries but only one in ten participants listed fluoride toothpaste as a means for prevention. Only one-third of participants had used toothpaste the previous day. There was a large, statistically significant difference in knowledge by location and those in Ratanakiri appeared to have a much poorer understanding of the causes of dental caries. There were significant differences in non-nutritious food consumption by location; those children in Phnom Penh consumed twice the amount of sweet drinks (including sweet milk beverages) compared with children from other regions. On the other hand, those children in the two outlying provinces of Ratanakiri and Kratie consumed around 1 packaged snack per day more than children in Phnom Penh.

**Table 3:** Oral health knowledge and behaviours by health knowledge and behaviours by location^a^

	Total N^d^ (column %)	Phnom Penh N (row %)	Kratie N (row %)	Ratinakiri N (row %)
Oral Health Knowledge				
Primary teeth are important	3116 (80.2)	834 (82.7)	1257 (79.8)^c^	1025 (78.9)^c^
Tooth decay is preventable	2671 (68.8)	784 (77.8)^bd^	1094 (69.4)^bc^	793 (61.0)^cd^
Tooth decay can be prevented by				
Tooth brushing	2304 (59.3)	666 (66.1)^bd^	937 (59.5)^bc^	701 (54.0)^cd^
Fluoride toothpaste	370 (9.5)	361 (35.8)^bd^	3 (0.2)^b^	6 (0.5)^d^
Avoiding sweet food	248 (6.4)	217 (21.5)^bd^	15 (1.0)^b^	16 (1.2)^d^
Visiting the dentist	927 (23.9)	9 (0.9)^bd^	594 (37.7)^bc^	324 (24.9)^cd^
Preventing in other ways	438 (11.3)	30 (3.0)^bd^	298 (18.9)^bc^	110 (8.5)^cd^
Tooth decay is caused by:				
Sugar	1794 (46.2)	834 (82.7)^bd^	959 (60.9)^bc^	1 (0.1)^cd^
Bacteria	37 (1.0)	7 (0.7)^bd^	3 (0.2)^bc^	27 (2.1)^cd^
Inherited/genetic	104 (2.7)	2 (0.2)^bd^	33 (2.1)^bc^	69 (5.3)^cd^
Other cause	1163 (30.0)	144 (14.3)^bd^	579 (36.7)^bc^	440 (33.9)^cd^
Brushed the child’s teeth yesterday	1847 (49.9)	494 (51.1)^bd^	705 (47.1)^bc^	648 (52.4)^cd^
Used toothpaste when brushing	1361 (36.8)	260 (26.9)^bd^	662 (44.2)^bc^	439 (35.5)^cd^
Mean number of sweet drinks/day	1.9 (SD2.6)	3.1 (2.3)^b^	1.5 (3.0)^bd^	1.4 (1.7)^d^
Mean number of packaged snacks/day	3.3 (2.7)	2.5 (1.7)^b^	4.0 (3.4)^bcd^	3.1 (2.4)^cd^

^a^N represents the number of participants who agree (eg. The number of participants to state that primary teeth are important) or who or list an item (eg. Tooth brushing) in response to open ended questions about oral health knowledge and behaviours

^b,c,d^ Means that in groups within the same row with a common super script letter have a statistically significant different (P = < 0.05;χ^2^ or *t*-test)

Overall one third (33.5%) of caregivers rated their child’s oral health as “good” or “excellent” with no significant differences across provinces. Among Phnom Penh children aged 2-years and older (*n* = 481), there was no difference in FIS scale and subscale scores by age group. Around one in ten families reported one or more impacts across the FIS domains (Table 4). No participant had a maximum score, however ‘floor effects’ were evident with 402 (84.2%) participants recording the minimum score of zero. Around one in ten families of children were categorised as having impacts across the FIS, and the emotions subscale appeared to be the most affected. Those children with one or more cavitated lesions and those children who had one or more pulpally-involved teeth had a seven-fold greater chance of realising an impact across the FIS.

**Table 4:** Prevalence of impacts across the Family Impact Scale and subscale scores for Phnom Penh participants

	Family Impact Scale	Activities Subscale	Emotions Subscale	Conflict Subscale
Gender				
Male	26 (10.4)^a^	6 (2.4)	23 (9.3)^a^	3 (1.2)^a^
Female	41 (16.5)	12 (4.8)	37 (14.9)	10 (4.0)
Age-group				
< 12-months	0 (0.0)	0 (0.0)	0 (0.0)	0 (0.0)
12–23 months	4 (10.0)	2 (5.0)	3 (7.5)	0 (0.0)
24–35 months	47 (14.0)	13 (3.9)	42 (12.5)	11 (3.3)
36–47 Months	16 (15.7)	3 (2.9)	15 (14.7)	2 (2.0)
Caries experience				
No carious lesions	5 (3.5)^b^	5 (3.5)	1 (0.7)^b^	1 (0.7)^b^
Any cavitated lesions	59 (22.4)^b^	13 (4.9)	56 (21.3)^b^	12 (4.6)^b^
Any pufa	10 (24.4)^a^	2 (4.9)	9 (22.0)^a^	2 (4.9)
Overall	67 (13.5)	18 (3.6)	60 (12.0)	13 (2.6)

^a^*P* < 0.05;χ^2^ test for differences in impacts by gender, presence of cavitated lesions, or pulpally involved lesion

^b^*P* < 0.005; χ^2^ test differences in impacts by presence of, or pulpally involved lesion

## Discussion

This study represents the first opportunity to examine ECC in a Cambodian context using an epidemiological survey; it confirms the findings of previous convenience samples in demonstrating that the caries experience of preschool children is severe, and affects a large proportion of both urban and rural children. Although the FIS is a well-recognized instrument for measuring OHRQoL, this study is also the first time that the FIS has been used in an epidemiological survey and it was able to demonstrate differences in impacts according to the presence of disease.

This study is unique in that there was an attempt to incorporate a participant reported indicator for caries disease experience. The instrument selected in this case was the FIS due to the fact that it had been previously validated for use in a Cambodian population. Possibly the Early Childhood Oral Health Impact Score might have been a better epidemiological tool for describing OHRQoL at population level, as reported by other authors [8]. However, the FIS has demonstrated that ECC is not just an issue for the affected children, but also their households; ECC in this study was associated with a higher prevalence of impacts on the family. It is also interesting to note that the ‘Family Emotions’ subscale appeared to be the most affected; families experienced feelings of being upset or guilty about the state of their child’s mouth. It appears that ECC can undermine the functioning of a household, particularly if members of the household experience additional stressors as a result of dental caries. For example if parents lose sleep or cannot attend work as a result of the symptoms of dental caries in their child.

The prevalence and severity of ECC in Cambodia appears to be similar to that observed in urban areas of the Lao Peoples Democratic Republic and the Philippines. In contrast, children in neighboring Thailand have benefited from a reduction in Early Childhood Caries in recent years as a result of a nationwide oral health strategy, and those in Singapore appear to have the lowest prevalence in the region due to favorable socio-demographic status and water fluoridation. Comparisons of ECC across the SEA region are however difficult given the variable sampling techniques and the fact that much of the data is now out of date [1]. The SEA Early Childhood Caries Forum identified the need for more recent and higher quality epidemiological data; therefore the aim of this paper was to present a description of ECC in a representative sample of Cambodian children, ahead of a more complex and multidisciplinary exploration of how dental caries is associated with a range of other behavioural, developmental, and nutritional factors.

The finding that one in six children have one or more pulpally-involved teeth by the age of 3-years is a remarkable statistic with wide-reaching implications in terms of disease burden. Around half of the lesions observed in the present study were only in the enamel, demonstrating an opportunity for intervention before cavitation occurs. If the non-cavitated lesions could be managed in the preschool age group using simple and non-invasive methods, then that would significantly reduce severity of the symptoms that are experienced by those children as they grow older. A reduction in caries experience might logically have a positive impact on a child’s ability to eat, sleep and learn well; future investigations will address this research question.

Some differences were observed in the prevalence and severity of ECC across provinces and this might partly be explained by the differences in consumption of non-nutritious high-sugar foods. Children in Phnom Penh had a higher consumption of sugary drinks, which are known to be the largest contributor of free sugars in children’s diets. In contrast, those children in more remote areas had a higher consumption of packaged snacks which, although being non-nutritious, contain less sugar than sugary drinks [11]. If those in the urban areas are consuming more sugar then it is also worth noting that approximately half of the lesions in the Phnom Penh sample were cavitated in contrast to just one-third of lesions in the two other provinces. This is the first epidemiological survey to provide a preliminary examination of the consumption of non-nutritious snacks among this age-group and the present findings provide some cause for alarm. WHO has recently updated its recommendations concerning the amount of free sugars which can be safely consumed [12]. However further investigations are needed in Cambodia to define sugar consumption both in terms of quantity and in terms of the associated social factors and behavioral factors that accompany that consumption.

High consumption of non-nutritious foods and their effect on health could create challenges for the Cambodian health system and workforce [12]. While sugar is recognized as the primary driver of the caries process, the impact of non-nutritious food consumption on other health conditions must also be considered. A better understanding of the complex relationship between the presence of carious lesions and the presence of other health conditions such as nutritional disorders is needed. Multi-sectorial initiatives should be implemented to reduce the consumption of non-nutritious foods in young children, and to support the Cambodian population to align with the WHO recommendation for sugar consumption; that just 5% of calorific energy intake should be sourced from ‘added sugar’ [11].

Despite the uniformly high sugar consumption, it does appear that differences in knowledge and behaviors may have moderated some of the disease experience of the preschool children when we compare the urban and rural samples. Those in Phnom Penh had a lower ratio of pulpally-involved teeth to cavitated lesions (PUFA ratio [9]) and this could possibly be explained by the higher proportion of caregivers in Phnom Penh who appeared to have a more favorable understanding of dental caries and the ways in which it can be prevented. The relationship between oral health literacy and dental caries experience is an investigation that could be explored as a way of influencing oral health behaviors.

The results of this study, presenting data on both the observable signs of dental caries together with evidence of impacts on the family unit, provides compelling evidence that urgent action is needed to address the ECC issue in Cambodia. Caries is an entirely preventable disease. A recent pilot study of the ‘Cambodia Smile’ intervention in Kampong Speu province demonstrated success in reducing dental caries among 2-year old children through the use of primary health care workers to deliver an oral health intervention [13]. Further validation of such low-cost public health interventions is needed in order to justify policy change and implementation of preventive programs on a wider scale.

## Conclusion

The paper presented descriptive data on ECC and highlighted the need for further multidisciplinary exploration of how dental caries interacts with other behavioral, developmental, and nutritional factors. The study makes it clear that preschool children in Cambodia suffer from a severe burden of dental caries which also has impacts on others in the household. There is strong evidence that ECC is a pressing issue for the Cambodian population and urgent action should be taken in order to reduce the burden of disease using evidence-based approaches.

## Data Availability

The datasets used and/or analysed during the current study are available from the corresponding author on reasonable request.

## Abbreviations

CAHENMS: Cambodia Health and Nutrition Monitoring Study
CNOHS: Cambodia National Oral Health Survey
ECC: Early childhood caries
FIS: Family impact score
OHRQoL: Oral-health-related quality-of-life
PUFA: The Pulpally involved, Ulcerated, Fistula and Abscess index
SEA: South East Asian
SEA-ECC: The South East Asian Index for Early Childhood Caries
